# Snail promotes an invasive phenotype in lung carcinoma

**DOI:** 10.1186/1465-9921-13-104

**Published:** 2012-11-17

**Authors:** Heta Merikallio, Taina Turpeenniemi-Hujanen, Paavo Pääkkö, Riitta Mäkitaro, Kaarteenaho Riitta, Sirpa Salo, Tuula Salo, Terttu Harju, Ylermi Soini

**Affiliations:** 1 Department of Internal Medicine, Respiratory Research Unit, University of Oulu and Oulu University Hospital, Oulu, Finland; 2 Clinical Research Center, Oulu University Hospital, Oulu, Finland; 3 Department of Oncology and Haematolgy, University Hospital of Oulu, University of Oulu, Oulu, Finland; 4 Department Pathology, University Hospital of Oulu, Oulu, Finland; 5 Oulu Center for Cell-Matrix Research, Institute of Dentistry, University of Oulu, Oulu, Finland; 6 Institute of Dentistry, University of Helsinki, Helsinki, Finland; 7 Department of Pathology and Forensic Medicine, University of Eastern Finland, Kuopio and Cancer Center of Eastern Finland, Kuopio, Finland

**Keywords:** Lung, Carcinoma, Snail, Claudin, MMP

## Abstract

**Background:**

Snail is a transcriptional factor which is known to influence the epitheliomesenchymal transition (EMT) by regulating adhesion proteins such as E-cadherin and claudins as well as matrix metalloproteases (MMP).

**Methods:**

To evaluate the functional importance of snail, a transciptional factor involved in EMT in lung tumors, we investigated its expression in a large set of lung carcinomas by immunohistochemistry. Expression of snail and effects of snail knockdown was studied in cell lines.

**Results:**

Nuclear snail expression was seen in 21% of cases this being strongest in small cell lung carcinomas (SCLC). There was significantly greater snail expression in SCLC compared to squamous cell or adenocarcinoma. Positive snail expression was associated with poor survival in the whole material and separately in squamous cell and adenocarcinomas. In Cox regression analysis, snail expression showed an independent prognostic value in all of these groups. In several cell lines knockdown of snail reduced invasion in both matrigel assay and in the myoma tissue model for invasion. The influence of snail knockdown on claudin expression was cell type specific. Snail knockdown in these cell lines modified the expression of MMP2 and MMP9 but did not influence the activation of these MMPs to any significant degree.

**Conclusions:**

The results show that snail plays an important role in the invasive characteristics of lung carcinoma influencing the survival of the patients. Snail knockdown might thus be one option for targeted molecular therapy in lung cancer. Snail knockdown influenced the expression of claudins individually in a cell-line dependent manner but did not influence MMP expressions or activations to any significant degree.

## Background

One biological function which is important in for the metastatic behaviour of tumors is epitheliomesenchymal transition (EMT). In EMT, epithelial tumor cells attain fibroblastic/myofibroblastic characteristics and in this way may be more capable of invading neighbouring structures and metastasizing [1,2]. It has been shown that during this transition there is a downregulation of several adhesion molecules such as E-cadherin. It is believed that the activation of transcription factors, such as slug, snail or twist is responsible for EMT and downregulation of these adhesion molecules in epithelial cells [1,2]. The activation of the transcription factors, on the other hand, can be influenced by several signalling molecules, such as transforming growth factor beta (TGFβ), bone morphogenetic protein (BMP), fibroblastic growth factor (FGF) or notch[3]. In addition to influencing EMT, these zinc-finger transcription factors are essential in the embryonic development where they regulate the development of mesodermal tissues [3].

The transcription factor snail is a member of the zinc-finger transcription factor family. Snail has been shown to be a mediator of EMT because it can down-regulate E-cadherin transcription and up-regulate the matrix metalloproteases (MMPs) such as MMP9 [4,5]. Snail has also been found to downregulate the expression of tight junction proteins occludin and claudins [1,6,7]. In this way snail regulates several genes which are involved in EMT. In addition, snail also influences apoptosis and cell movement and in this way may also play a role in the spread of the tumor [3].

Matrix metalloproteases (MMPs) are associated with tumorigenesis and EMT. They are zinc dependent endopeptidases that are capable of degrading all kinds of extracellular matrix proteins [8,9]. This enables the tumor cells to migrate, invade and spread into neighbouring tissues. MMP 2 and MMP 9 are gelatinases which can disturb cell adhesion by processing the components of cell-cell and cell-extracellular matrix contacts [10].

Different expression of claudins has been found in different types of epithelial tumors and also in mesothelial and endothelial neoplasms [11,12]. While endothelial neoplasms strongly express claudin 5, the expressions of other claudins are more varied in epithelial and mesothelial tumors [11,12]. For instance in prostate carcinomas there are intense expressions of claudins 3 and 4, while claudin 2 expression is less intense [13]. In lobular carcinoma of the breast and in diffuse carcinoma of the stomach, claudin expression is downregulated compared to the invasive ductal type or intestinal carcinoma of the stomach [14,15]. The expression of claudins may also vary depending on the grade or stage of the tumors, for instance in breast or esophageal carcinoma [15,16]. Thus the claudins also play a crucial role in the clinical behaviour of tumors.

In this study, we investigated the expression of snail in a large set of lung tumors and the association of selected tight junction proteins and matrix metalloproteinases with snail expression. The effect of snail knockdown to tumor cell invasion, and claudin and MMP2 expression was studied in cell lines. Our goal was to study the importance of snail in lung carcinoma spread and whether claudins or MMP 2 and 9 would be mediators on snail induced EMT in lung carcinomas.

## Methods

### Materials studied

The study material consisted of 136 squamous cell carcinomas, 108 adenocarcinomas, 21 small cell carcinomas, 11 large cell carcinomas and 13 carcinoid tumors. Material was collected from the files of the Oulu University Hospital, Oulu, Finland. The diagnosis of the cases was based on the WHO classification [17] of lung and pleural tumors. The presence of metastases was determined at the time of the operation.

### Immunohistochemistry for claudins, snail and MMPs

Immunohistochemistry for claudins was performed as described earlier Merikallio et al. (2011) [18]. For the immunohistochemical staining of MMPs, four-micrometer sections were cut on slides coated with poly-L-lysine (Sigma-Aldrich, St Louis, MO, USA) and incubated overnight at 37°C. The slides were deparaffinized and hydrated and treated with 0.5% pepsin (2000 FIP-U/g, Merck, Darmstadt, Germany) at 37°C. Endogenous peroxidase activity was blocked by incubating the slides in 3% hydrogen peroxide in absolute methanol and non-specific binding was blocked with 10% goat serum. Mouse monoclonal antibody (24 μg/ml), for MMP 2 (CA-4001, Diabor, Oulu, FI) and mouse monoclonal antibody to MMP 9 (10 μq/ml) (GE-231 Diabor, Oulu, FI) were used as primary antibodies. The slides were incubated with primary antibody at RT overnight after which the Histostain bulk kit was used (Zymed Laboratory Inc., San Fransisco, Ca, USA). The sections were counterstained with haematoxylin and mounted with Immuno-mount (Shanon Inc., Pittsburgh, PA, USA). For negative controls, the primary antibody was replaced by mouse non-immune IgG or PBS.

For snail the staining procedure was as follows. The sections were first deparaffinized and rehydrated in graded alchohol, then heated in microwave oven for 2 × 5 min in Tris-EDTA buffer (pH 9.0), and incubated in Tris-EDTA buffer for 20 min. After being washed two times for 5 min in phosphate buffered saline (PBS), endogenous peroxidase was blocked with 5% hydrogen peroxide for 5 min. Non-specific binding was blocked with 1.5% normal serum in PBS for 35 min at RT (room temperature). The sections were incubated overnight at 4°C with the primary antibody for SNAI1 (monoclonal anti-SNAI1 antibody was a kind gift from the Department of Anatomy, University of Helsinki, Helsinki, Finland) 1: 1000 dilution (Takkunen et al. 2006) and incubated with the biotinylated secondary antibody and avidin-biotinylated peroxidase complex (ABC Vectastain Elite Kit, Vector Laboratories, Burlingame, CA, USA). The colour was developed with diaminobenzidine (DAB) (Sigma-Aldrich). The slides were counterstained with haematoxylin, and mounted with Depex (BDH, Poole, UK). Negative control stainings were carried out by substituting non-immune mouse serum and PBS for the primary antibodies.

The evaluation of stainings was performed by two experienced pathologists for snail and claudins (RK, YS) and for MMPs pathologist (YS) and an experienced investigator (TTH).

For claudins, localisation of reaction product to cell membrane and the characteristics of the immunostaining were recorded and the staining was quantified as follows

0 = negative, 1 = less than 25% of positivity, 2 = 25–50% positivity, 3 = 50–75% positivity and 4 = over 75% positivity.

In the evaluation of the associations the results were divided into weak (0,1) and strong (2–4) positivity.

The immunostainings for MMPs were first assessed by dividing them into five groups (0 = negative, 1–25% = weak, 25–50% = moderate, 50–75% = strong, over 75% = very strong). For the final analysis, the cases with less than 25% of cells were considered as negative and the data was divided in two groups; negative (< 25%) and positive (≥ 25%). In cases of disagreement in the staining assessment, a consensus decision was reached by reviewing the case once more.

With respect to snail, nuclear expression for tumor cells was calculated in the array samples thus giving a value of positivity per array area. In each case, two separate array samples from each case were studied. We also recorded positivity or negativity based on the presence of snail positive nuclei in tumors.

### Cell Lines

Human non-malignant bronchial BEAS-2B cells which are SV40 transformed cells and human lung carcinoma SK-MES-1 and SK-LU-1 cell lines were obtained from American Type Culture Collection (Rockville, MD). Cells were cultured according to the instructions of the American type culture collection.

### Snail knockdown cell lines

Lentiviruses were produced in Phoenix-GP packaging cells (PhGP-cells). PhGP-cells were transfected with lipofectamine 2000 (Invitrogen, Carlsbad, CA, USA). Cells were given low glucose medium just before transfection. For transfection, a mix of Optimem (Invitrogen, Carlsbad, CA, USA) was prepared containing lipofectamine 2000, the desired retroviral vector Slug 865 (Sigma-Aldrich, St.Louis, MO, USA) and core protein plasmids (REV, pVSV-G and Gag/Pol). The mix was added to the cells and the 24 hours post transfection medium was removed and changed to fresh medium for virus-collection. Viruses were collected three times. BEAS-2B, SK-LU-1 and SK-MES-1 cells were infected with fresh virus-stocks. Polybrene (Sigma-Aldrich, St.Louis, MO, USA) was added in each infection to enhance the efficiency of the infection. After the infections, the infected cells were separated from the normal cells by antibiotic selection. Puromycin (Sigma-Aldrich, St.Louis, MO, USA) was added to the normal mediums of the cell lines to exterminate cells that were not infected.

### RT-PCR analyses

RT-PCR and result analysis were carried out as described before [18].

### Zymograms

Cells were grown in serum free media and it was collected from the cells, centrifuged and the supernatants were and concentrated with 10 000 cut-off concentration tubes (Millipore) to final volume of 150 μl. Proteins were measured with DC protein assay-kit (BIO-RAD) and 3 μg of each sample were applied to 1,5 mm 10% SDS polyacrylamide slab gels casted in the presence of 1 mg/ml fluorescently (2-methoxy-2,4-diphenyl-3-[2H]furanone) labeled gelatin (Fluka Ronkonkoma, NY) according to the method described by O’Grady et al. 1984. After electrophoresis, SDS was removed by 2.5% Triton X-100 to reactivate the gelatinases. Gels were incubated in 50 mM Tris–HCl buffer (pH 7.8, 150 mM NaCl, 5 mM CaCl_2_, 1 M ZnCl_2_) overnight at 37°C. A set of corresponding gels were incubated overnight at 37°C with 10 mM EDTA in 50 mM Tris–HCl to inhibit the metalloproteinase activities. The gelatin degradation was visualized under long-wave ultraviolet illumination followed by 0.5% Coomassie Blue R-250 staining of the gels. The gels were photographed and cleavage rates of gelatin were estimated by determining the rates of disappearance of gelatin by densitometric scanning of the photographed gels.

### Invasion experiments

Invasion of the cells were studied by two different assays, the gel matrix assay and myoma organotypic model. Both original and blocked cell lines were studied in these models. As the gel matrix assay, the Culturex BME cell invasion assay from Trevigen (Gaithersburg, MD) was used and carried out according to the instructions of the manufacturer.

In the myoma organotypic assay 400 000 cells were cultured on the myoma tissue for ten days and fixed with formalin for the histochemistry and immunohistochemistry (Nurmenniemi *et al*. 2009). The 4 μm thick slides were stained with haematoxylin and eosin and with anti-human cytokeratin (DakoCytomation, Glostrup, Denmark) to evaluate the distance for the maximal invasion depth (the distance from the lower surface of the non-invasive cell layer to the deepest invaded cell) by light microscopy.

### Statistical analysis

Comparison between groups, was made by Mann–Whitney U-test, Kruskall-Wallis test and t-test The significance of the associations was determined by Fisher’s exact probability test and correlation analysis. Survival data were studied by Kaplan-Meier method and Cox regression model. Probability values ≤ 0.05 were considered significant.

### Ethical considerations

The study was approved by the ethical committee of Northern Ostrobothnia Hospital District. The survival data was obtained from the Finnish Cancer Register after receiving permission from the Ministry of Health and Social Welfare and the National Supervisory Authority for Welfare and Health.

## Results

### Expression of snail in lung tumors

**Figure 1:**
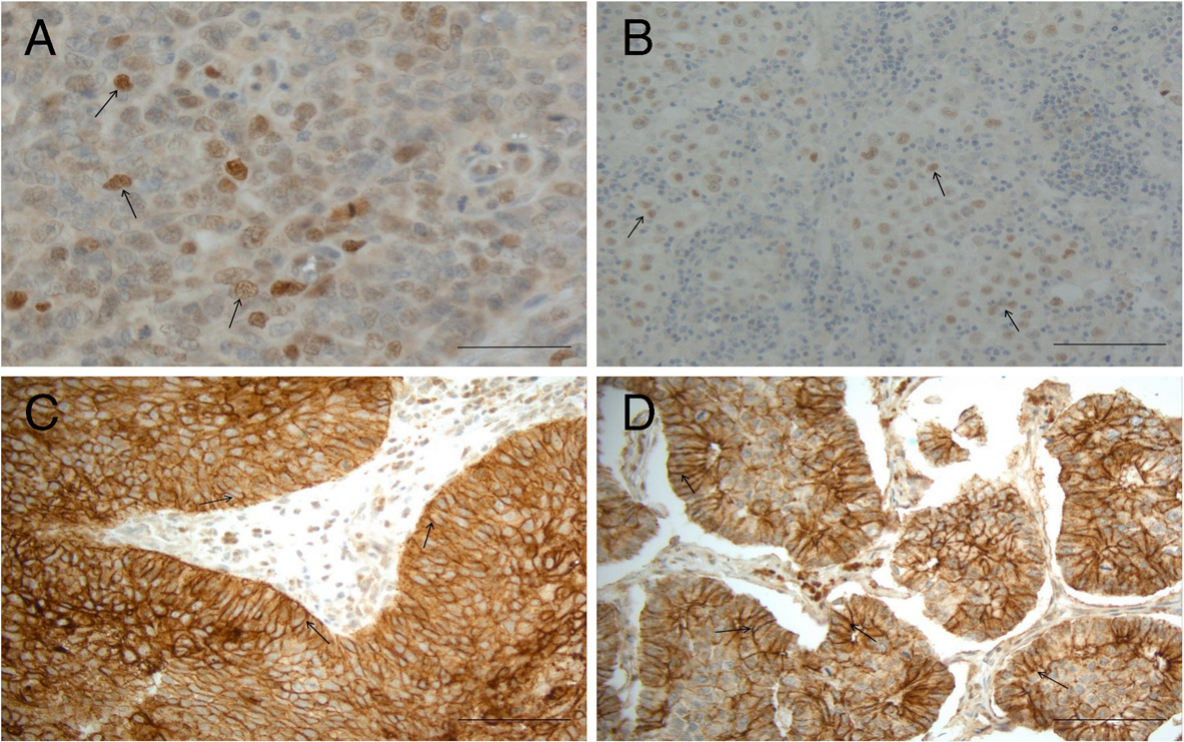
**Snail and claudin immunoreactivity in different carcinomas.A**. in a small cell carcinoma of the lung, intense nuclear snail positivity, **B**. in a large cell carcinoma of the lung, nuclear positivity for snail, **C**. in a squamous cell carcinoma, membrane bound positivity for claudin 1 can be detected. The stromal tissue is negative and **D**. in an adenocarcinoma of the lung, membrane bound positivity for claudin 3 can be seen. Scale bar is 200 μm in the figures. Positive examples are shown in figure by arrows (→).

Nuclear snail positivity was found in (63/279) 22.6% of the cases (Table 1).The distribution of snail positivity in different histological types of tumors is given in Table 1. We also assessed the number of snail positive cells quantitatively in the array samples. The mean number of positive nuclei in tumor cells in array samples was 6.5 (range 0–703). In squamous cell carcinomas of the lung, the mean number of positive tumor cells in array samples was 2.5 (range 0–58) and in adenocarcinoma it was 8.1 (range 0–624). In small cell carcinoma it was 31.2 (range 0–326) and in large cell carcinoma 19.4 (range 0–183). (Figure 1A and 1B). In contrast carcinoid tumors showed no positivity. In primary lung tumors, the difference in the amount of nuclear positivity was significant between squamous cell carcinoma and small cell carcinoma (p = 0.047), and between adenocarcinoma and small cell carcinoma (p = 0.013).

**Table 1:** Nuclear snail expression in tumor cells

	**Tumor cells**	
**Histology**	**Negative**	**Positive**
Squamous cell carcinoma	95	31
Adenocarcinoma	88	20
Small cell carcinoma	12	9
Large cell carcinoma	8	3
Carcinoid tumors	13	0

Nuclear (p = 0.51) snail expression did not display any association with the presence of metastases or with the grade of the tumors.

### Impact of snail expression on the patient′s prognosis

**Figure 2:**
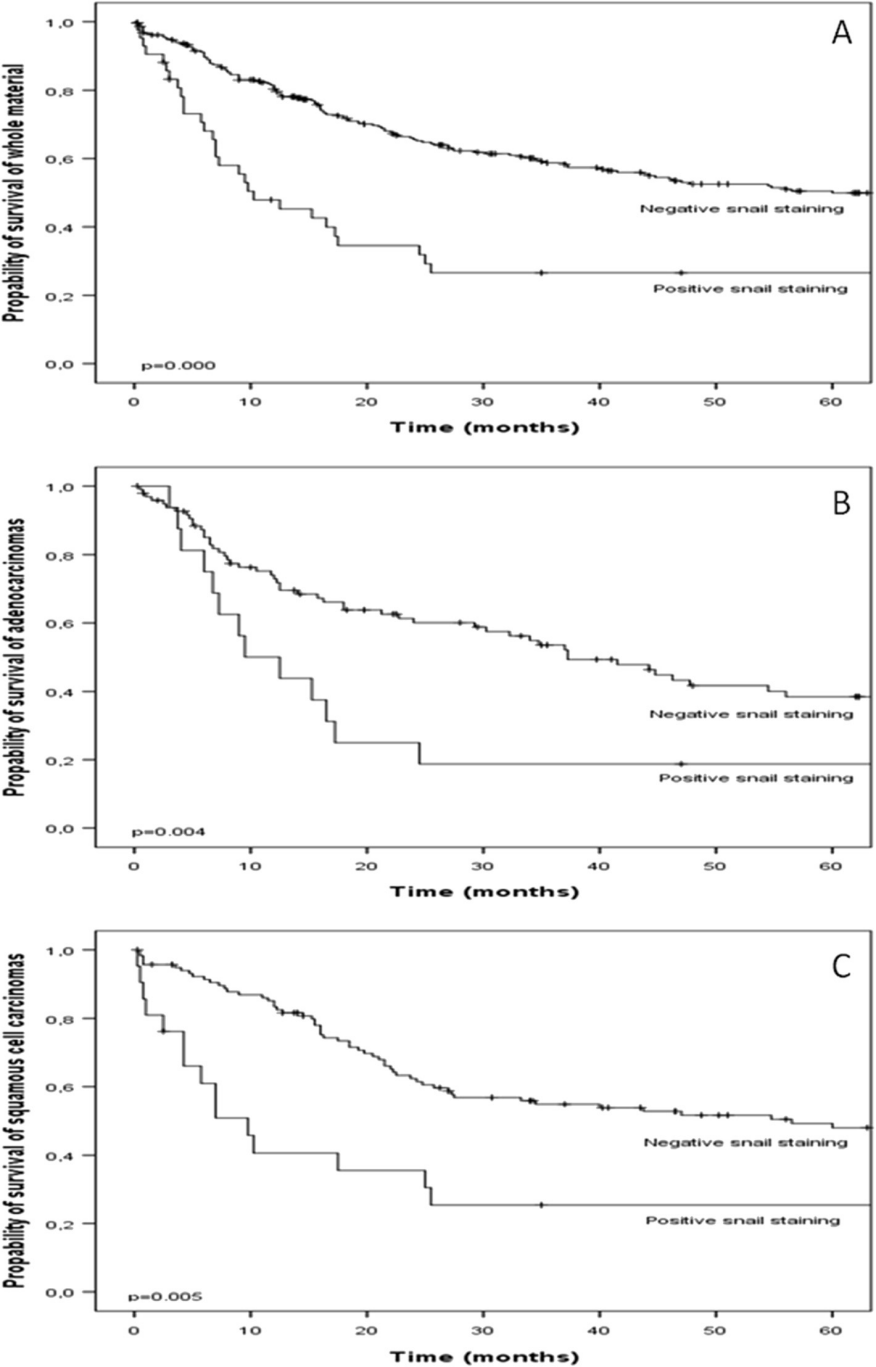
**Kaplan-Meier estimates of survival.A**. survival association of snail expression in whole material (p = 0.000), **B**. in adenocarcinomas (p = 0.004) and **C**. in squamous cell carcinomas (p = 0.005).

Patients with negative snail expression lived longer than patients with positive snail expression in the whole material (p ≤ 0.001) (Figure 2A). This was also seen (Figure 2B and 2C) in two major subgroups of lung tumors, in adenocarcinomas and squamous cell carcinomas (p = 0.004 and p = 0.005, respectively). In the whole material, those patients who exhibited strong snail immunoreactivity had only half the life expectancies than patients without snail immunoreactivity (p = 0.001). According to Cox multivariate regression analysis, snail expression had an independent prognostic value in the whole tumor group and separately in squamous and adenocarcinoma (Table 2). The size of tumour with snail expression increased the risk of shorter survival in all carcinomas. The presence of metastases did not change the risk factor for shorter survival. Smoking did not change the hazard ratio.

**Table 2:** Cox multivariate regression analysis of snail expression in the whole tumor group and separately in squamous cell and adenocarcinoma

			**95% Cl for hazard ratio**	
**Snail**	**Significance**	**Hazard ratio**	**Lower**	**Upper**
All cases	0.001	2,074	1,326	3,245
Adenocarcinomas	0.037	2,07	1,047	4,095
Squamous cell carcinomas	0.002	2,523	1,423	4,475

### Relationships between expression of claudins and snail in lung tumors

The expression of claudins 1, 2, 3, 4, 5 and 7 in lung tumors for the material has been presented previously [18]. In cases where claudin 1 expression was low, nuclear snail expression was high as compared to cases with high claudin 1 expression (15.5 vs 2.3 nuclei/array, p = 0.023). In cases where claudin 3 expression was low, nuclear snail expression was also higher compared to those cases with high claudin 3 expression but the association did not reach statistical significance (12.6 vs 4.0 nuclei/array, p = 0.14).

Claudin 5 expression was upregulated when there was negative snail expression (p = 0.034) and this tendency was also seen with claudin 7 (p = 0.062). There were no significant associations between snail and other claudins.

There was no significant correlation between snail expression and the expression of MMP2 (p = 0.057) or MMP9 (p = 0.102) in the tumor material. MMP2 and 9 associated significantly between each other (p < 0.001).

### Effect of snail knockdown of claudin expression in cell lines

**Figure 3:**
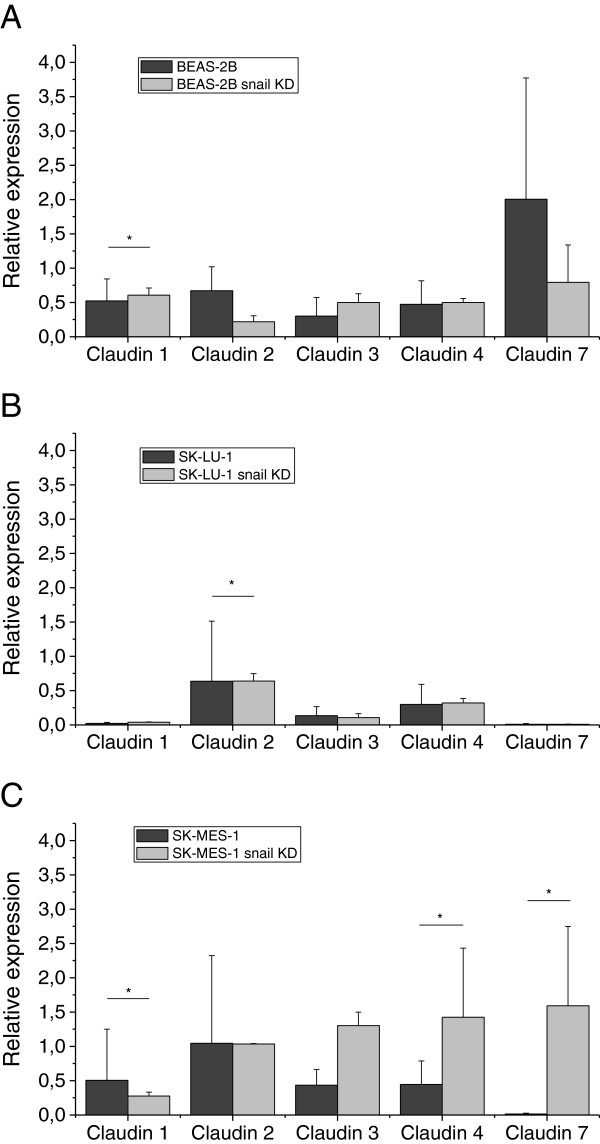
**Claudin mRNA expression 1, 2, 3, 4 and 7 in normal cell lines and snail knockdown cell lines.A**. The difference between normal and snail knockdown BEAS-2B cell lines was significant for claudin 7 (p = 0.001) and near to significant for claudin 4 (p = 0.052), **B**. between the SK-LU-1 cell line and snail knockdown cell line, there were statistically significant differences in claudin 2 (p = 0.026) and **C**. The difference between normal and slug knockdown SK-MES-1 cell lines was significant for the expressions of claudin 1 (p = 0.005), claudin 4 (p = 0.011) and for claudin 7 (p = 0.005). Nearly statistically significant difference was seen for claudin 2 (p = 0.059).

**Figure 4:**
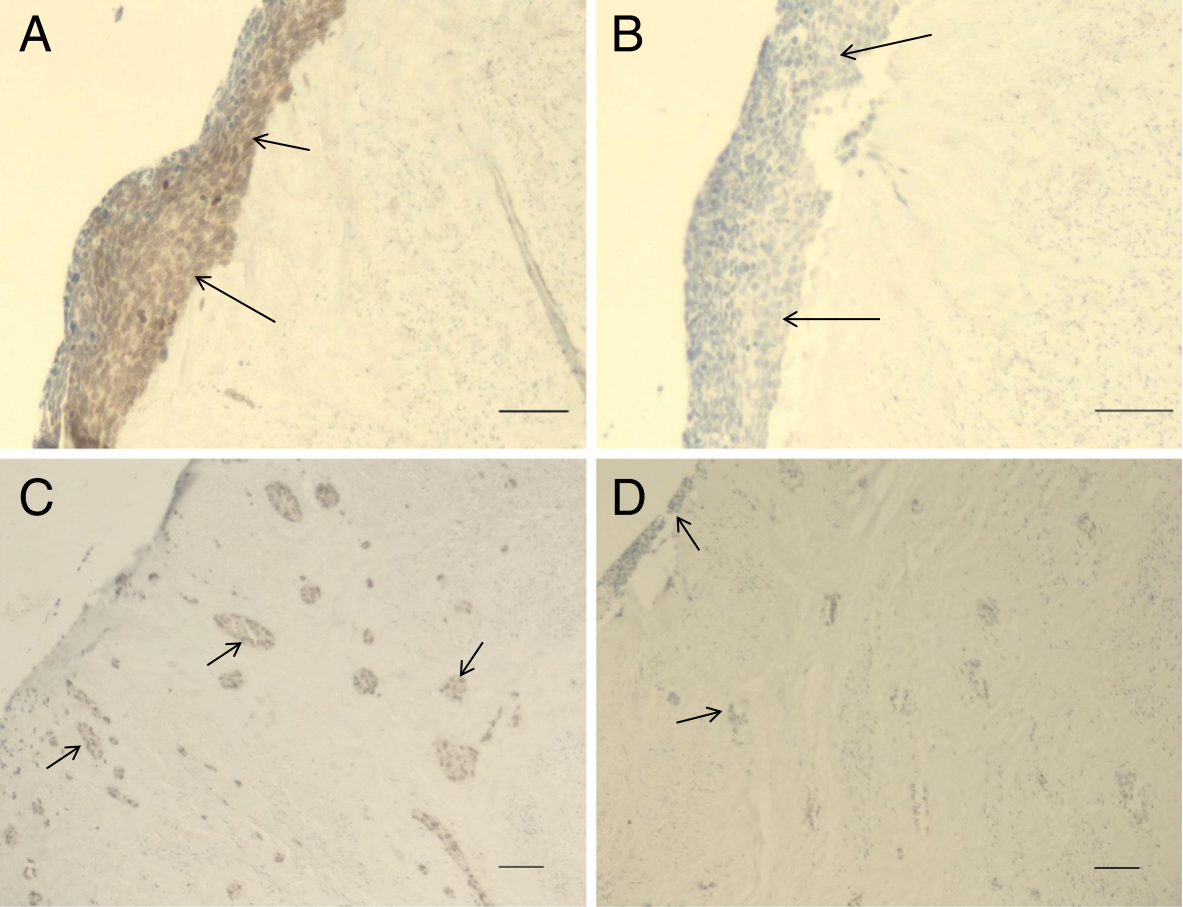
**Snail immunoreactivity in carcinoma cell lines.A**. SK-LU-1 carcinoma cell line, **B**. SK-LU-1 snail knockdown cell line, **C**. SK-MES-1 cell line and **D**. SK-MES-1 snail knockdown cell line. Scale bars are 200 μm. Snail positivity is showed with arrows (→) in figures **A** and **C**. lack of snail expression is pointed with arrows (→) in figures **B** and **D.**

Surprisingly, snail knockdown in BEAS-2B cells decreased the expressions of claudins 2 and 7 (p = 0.001). Snail knockdown did not alter greatly the expression of claudins in the malignant SK-LU-1 cell line. The other malignant cell line, SK-MES-1, the snail knockdown did not alter claudin 2 expression, but claudin 3, 4 (p = 0.011) and 7 (p = 0.005) mRNA expression increased in knockdown line. Compared to the other malignant cell line, SK-LU-1, the claudin 1 mRNA expression was decreased in snail knockdown line (p = 0.005) (see Figure 3). Snail protein expression disappeared in knockdown carcinoma cell lines (Figure 4).

### Expression of MMP2 and MMP9 proteins *in vitro*

**Figure 5:**
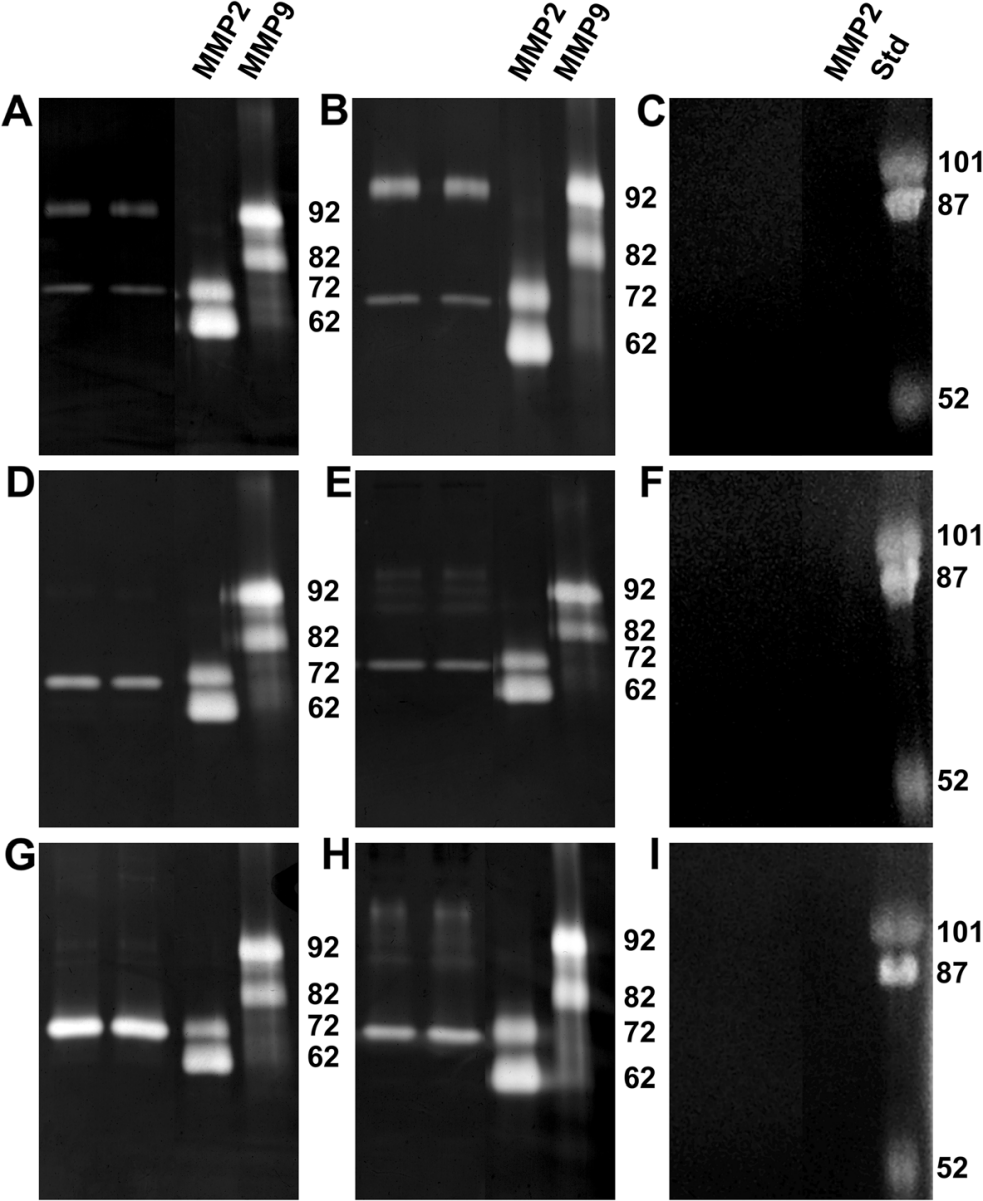
**Zymograms of the cell lines.** MMPs were studied from the medium from **A**. BEAS-2B cells, **B**. BEAS-2B snail knockdown cells, **C**. EDTA treated gelatin gels of BEAS-2B and knockdown cell lines, **D**. SK-LU-1 cells, **E**. SK-LU-1 snail knockdown cells, **F**. EDTA treated gelatin gels of SK-LU-1 and knockdown cell lines **G**. SK-MES-1 cells, **H**. SK-MES-1 snail knockdown cells and **I**. EDTA treated gelatin gels of SK-MES-1 and knockdown cell lines. MMP standards are in right side of the zymogramms. 92 kDa is proMMP9, 82 kDa is active MMP9, 72 kDA is proMMP2 and 62 kDa is active MMP2.

Original cell line BEAS-2B, produced proMMP 2 and proMMP 9 proteins (Figure 5A). Knockdown cell line produced more proMMP 9 protein than original cell line. The expression of proMMP 2 did not change in the knockdown line compared to the original cell line (Figure 5A and 5B). In malignant cells proMMP 2 was expressed, but not MMP 9 proteins (Figure 5D and 5G). Both malignant knockdown cell lines produced various forms of the proMMP 9 protein (Fig 5E and H). Knockdown cell lines produced also proMMP 2 protein. Gelatinolytic activities were studied and they disappeared after EDTA treatment of the gels (Figures 5C, 5F and 5I).

### Invasion experiments

**Figure 6:**
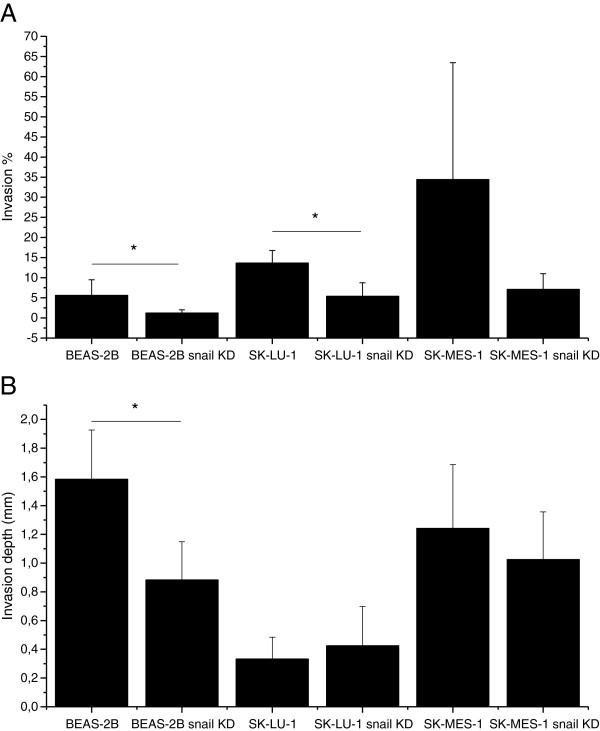
**Invasion assay results.A**. Gel matrix invasion assays results for BEAS-2B (p = 0.039), SK-LU-1 (p = 0.004) and SK-MES-1 cells and **B**. Myoma organotypic invasion model results. Invasion depth in normal and snail knockdown cell lines BEAS-2B (p = 0.003), SK-LU-1 and SK-MES-1.

The invasion properties were reduced in snail knockdown cells in all cell lines studied and by both invasion assay models in use. In the gel matrix assay (Figure 6A), the invasion properties of SK-MES-1 and SK-LU-1 were increased as compared to the situation in the BEAS-2B cells. All knockdown cell lines were less invasive than the original cell lines. The SK-MES-1 cell line was more invasive than SK-LU-1, this could be demonstrated in both assays. In the myoma invasion assay, the results were similar in BEAS-2B cells and SK-MES-1 cells i.e. snail knockdown cells were less invasive, in BEAS-2B cells invasion depth difference was statistically significant (p = 0.003) (Figure 6B). SK-LU-1 cells were more invasive than snail knockdown cell line in gel matrix assay (p = 0.004) (Figure 6A). Curiously, the SK-LU-1 snail knockdown cell line was slightly more invasive than original cell line in the myoma invasion assay (Figure 6B), but the difference was not statistically significant.

## Discussion

In this investigation, we studied the expression of snail in a large set of tumors consisting of histologically different primary lung carcinomas. The expression of snail was compared to the expression of claudins 1, 2, 3, 4, 5 and 7 and to the expression of MMPs 2 and 9 and to the clinical parameters in these tumors. We also studied the mRNA expression of snail in three cell lines, a transformed non-neoplastic bronchial cell line (BEAS-2B) and two neoplastic cell lines (SK-LU1, SK-MES1), determined their invasion properties by snail knockdown and the influence of snail knockdown on the expression of claudin mRNA expression as well as the expression and activation of MMPs 2 and 9.

Snail is a transcriptional factor which is known to influence the epitheliomesenchymal transition (EMT) and in this way it can influence the invasiveness of tumors. In skin squamous cell carcinomas of the mouse downregulation of snail caused to retarded growth and invasiveness of the cancer cells [19] and, inversely, expression of snail was associated with poor prognosis and metastatic potential of tumors in ovarian or head and neck carcinomas [20,21]. This is in line with our findings. In invasion experiments, knockdown of snail in the studied cell lines clearly retarded the invasion both in the collagen matrix assay and in the myoma tissue assay which is in line with the putative function of snail in promotion of EMT. Furthermore, expression of snail was associated with poor prognosis in the whole clinical lung tumor material and in squamous cell carcinoma and adenocarcinoma patients and its expression had an independent prognostic value. According to these results, snail expression appears to be significant for the spread of lung tumors which especially can also be seen separately in lung squamous and adenocarcinomas, the two main histological types of lung carcinomas.

Snail is known to downregulate the expression of E-cadherin and cytokeratin 18 and to upregulate the expression of vimentin and thus its expression leads to a mesenchymal phenotype of the tumor cells while the cohesion of the cells at the same time decreases [21,22]. In our tumor material there was an inverse association between the expression of snail and the expressions of claudins 1 and 5, and a similar tendency was observed for claudins 3 and 7. In cell line studies, knockdown of snail increased the expression of claudins 3, 4 and 7 mRNA in the SK-MES1 cell line while no effect was seen in the SK-LU1 cell line. Paradoxically, snail knockdown decreased the expression of claudin 1 mRNA in the SK-MES1 cell line and increased the expressions of claudins 2 and 7 mRNA in the BEAS-2B cell lines. This kind of discrepancies may be due to different environments in *in vivo* and *in vitro* conditions which could change the response of cells to snail. It has been shown that tumor stroma has a significant impact on EMT and may co-ordinate cellular responses in tumor spread. Different tumor cell lines may also show different responses depending on individual cell kinetics although the main reaction between the expression of snail and claudins would be that the former would decrease the expression of at least some claudins. In previous studies, overexpression of snail and slug decreased the expression of claudin 1 mRNA in Madin Darby kidney epithelial cells [7]. Claudin 3, 4 and 7 expressions were also shut down by snail in mouse epithelial cell lines [1].

In addition to being important in EMT and tumor invasion, snail also plays a role in embryonic development and it also influences apoptosis, angiogenesis and MMP9 expression, factors which are all important in tumor growth [3,23]. In our tumor array material, no significant association was found between snail and the expressions of MMP2 or MMP9, propably due to the small number of snail positive cases. In cell line studies, snail did not influence the expression or activation of MMP2 or MMP9 to a significant degree. This is in contrast to the results of some studies indicating induction of MMP9 or MMP2 by snail in tumors but this kind of effect may be cell line specific [9,24]. However, we could not observe any relation between snail and these MMPs in the array material with all results indicating that there was no evident association between MMP2 and 9 and snail in lung carcinoma.

We observed that snail was expressed in 22.6% of tumors although its expression varied depending on the histology of the tumors. The nuclear tumor-cell-associated expression of snail was strongest in small cell carcinoma of the lung, a tumor type which is one of the most aggressive lung tumors. On the other hand, tumor-cell-associated nuclear snail expression was not found in carcinoid tumors of the lung, also indicating that there is a link between such nuclear snail expression and biological behaviour of tumors.

## Conclusions

In summary we have studied the expression of snail *in vivo* and *in vitro* and correlated the findings with expression of claudins 1, 2, 3, 4, 5 and 7 and matrix metalloproteinases 2 and 9. Snail appears to be important in the invasive nature of lung carcinomas which is shown by its impact on patient survival as well as *in vitro* invasion assay experiments. While snail expression was inversely associated with some claudins the results were somewhat different in the histological data and cell line experiments. In conclusion, however, a part of snail’s impact on EMT seem to be mediated through downregulation of claudin expression although the influences may vary depending on the individual cell lines. According to our results, snail did not appear to have any effect on the expression of MMP2 or 9 in lung tumors although both of these factors surely act in concert with snail promoting in the spread of lung carcinomas.

## Abbreviations

EMT: Epitheliomesenchymal transition; MMP: Matrix metalloprotease; SCLC: Small cell lung carcinoma; NSCLC: Non-small cell lung carcinoma; TGFβ: Transforming growth factor beta; BMP: Bone morphogenetic protein; PBS: Phosphate buffered saline; DAB: Diaminobenzidine; RT-PCR: Real time polymerase chain reaction; mRNA: Messenger ribonucleic acid.

## Competing interests

The authors declare that they have competing interests.

## Authors' contributions

HM, THH, TS, RK and YS were involved in the conception, hypotheses delineation, anddesign of the study. HM, THH, RM, PP, SS, RK, YS and TH contributed to the acquisition of the data and the analysis and interpretation of such information. All authors participated in writing the article and had substantial involvement in its revision prior to submission.
